# The Effects of Tai Chi Chuan Versus Core Stability Training on Lower-Limb Neuromuscular Function in Aging Individuals with Non-Specific Chronic Lower Back Pain

**DOI:** 10.3390/medicina55030060

**Published:** 2019-03-03

**Authors:** Liye Zou, Yanjie Zhang, Yang Liu, Xiaopei Tian, Tao Xiao, Xiaolei Liu, Albert S. Yeung, Jing Liu, Xueqiang Wang, Qing Yang

**Affiliations:** 1 Lifestyle (Mind-Body Movement) Research Center, College of Sports Science, Shenzhen University, Shenzhen 518060, China; liyezou123@gmail.com; 2 Health and Exercise Science Laboratory, Institute of Sports Science, Seoul National University, Seuoul 08826, Korea; elite_zhangyj@163.com; 3 Department of Kinesiology and Program in Neuroscience, Indiana University Bloomington, Bloomington, IN 47405, USA; YL82@indiana.edu; 4 Department of Physical Education, Qiannan Normal University for Nationalities, Guizhou 558000, China; tianxiaopei1986@163.com; 5 College of Mathematics and Statistics, Shenzhen University, Shenzhen 518060, China; taoxiao@szu.edu.cn; 6 College of Chinese Martial Arts, Beijing Sports University, Beijing 100084, China; liuxiaolei99@hotmail.com; 7 Depression Clinical and Research Program, Massachusetts General Hospital, Harvard Medical School, Boston, MA 02114, USA; ayeung@mgh.harvard.edu; 8 Department of Martial Arts, Shanghai University of Sport, Shanghai 200438, China; 9 Department of Sport Rehabilitation, Shanghai University of Sport, Shanghai 200438, China; qiang897@163.com

**Keywords:** exercise, Tai Chi, lower back pain, neuromuscular function, muscular strength

## Abstract

*Objectives*: For this paper, we aimed to investigate the effects of Tai Chi Chuan (TCC) versus the Core Stability Training (CST) program on neuromuscular function (NF) in the lower extremities among aging individuals who suffered from non-specific chronic lower back pain (NLBP). Regarding the design, during a 12-week intervention, a single-blinded randomized controlled trial was used to compare two intervention groups with a control group on the parameters of NF. *Methods*: Forty-three Chinese community-dwellers were randomly assigned into two intervention groups (three sessions per week, with each session lasting 60 min in TCC and CST) and a control group. The patient-based Visual Analogue Scale (VAS) was used to measure the level of perceived pain, while parameters of NF as primary outcomes were measured by the Biodex System 3 Isokinetic Dynamometer. *Results*: For the knee joint, we observed significant differences in the endurance of left extension at a speed of 60°/s: (1) between TCC and control groups (*p* < 0.01); (2) between CST and control groups (*p* < 0.01). For the ankle joint, significant differences between CST and control groups were observed on the peak torque of left dorsiflexion (*p* < 0.05) and the endurance of the left plantar flexion at a speed of 60°/s (*p* < 0.05). In addition, we observed a significant difference between TCC and control groups in the endurance of the right plantar flexion (*p* < 0.05). *Conclusions*: Chen-style TCC and CST were found to have protective effects on NF in aging individuals with NLBP, while alleviating non-specific chronic pain.

## 1. Introduction

Lower back pain is a common musculoskeletal injury, which can be caused by overload/incorrect movement patterns at work, during exercise or other accidents [1]. According to the National Institute of Health, roughly 80% of adults experience lower back pain in their lifetime [2]. Data from the 2015 National Health Interview Survey has indicated that the annual average population with lower back pain was over 140 million [3]. More than 60 million adults over 45 years old currently have or have had, severe lower back pain [3]. Non-specific lower back pain is a type of back pain where the cause cannot be specifically determined, such as tumors, spine fractures or infections [4]. Non-severe lower back pain that is acute or sub-acute normally lasts from a couple of days to weeks and can be recovered from with proper rest and care [4]. However, pain that lasts longer than 12 weeks is categorized as chronic and around 20% of patients have acute back pain which develops into chronic back pain [4]. 

Neuromuscular function plays a critical role in performing everyday activities, as well as participating in physical activity or exercise programs to maintain well-being [5]. However, studies have found that non-specific chronic lower back pain (NLBP) can affect patients’ neuromuscular functioning [6,7]. These functional impairments were associated with reduced walking performance, as indicated by slower walking speed, jerky and awkward movement patterns and reduced coordination [8]. In addition, individuals with NLBP were found to experience social difficulties and had an avoidance of work, ultimately influencing their quality of life [9]. Whether or not they chose to seek treatment for NLBP varied according to the patient’s age, gender, willingness and several other related factors. Forms of treatment also varied from home-based self-care, to physical therapy, to surgery [9]. NLBP can cause socioeconomic burdens, not only to patients who are suffering from it but also to the medical system of the society that they are in [10]. Therefore, an ideal intervention strategy that is readily accessible, self-administered and cost-efficient is urgently needed. 

A recently published review paper on NLBP demonstrated that exercise may have neuromuscular protective effects, which may help to help broaden our understanding of methods to relieve lower back pain among this vulnerable population [11]. Among these studies, core stabilization training (CST) is becoming increasingly popular as a form of treatment for individuals with NLBP [12]. A recent meta-analysis paper by Wang et al. [12] indicated that this type of specifically designed training program is an efficient way to help reduce pain and disability, as well as to improve physical functioning. Furthermore, individuals with NLBP who practiced Pilates, a type of core stability exercise, were reported to have reduced pain and improved physical function, ultimately leading to better quality of life [13,14]. Another core stability exercise intervention study using a Swiss ball demonstrated that perceived disability decreased in this population, with improved neuromuscular function [15]. 

Tai Chi Chuan (TCC) is one of the types of traditional Chinese internal exercises that has provided numerous health benefits [16,17,18]. Studies conducted on different patient populations have found that TCC can help improve physical performance, reduce symptom-related pain and improve quality of life [19,20,21,22]. Previous studies also found that with practice of TCC, individuals with lower back pain had improved outcomes on pain and disability [23]. TCC is a type of exercise that not only involves physical movement but also engages mindfulness meditation during its practice [24,25,26]. Studies have demonstrated that even with mindfulness meditation, patients with lower back pain may observed improvements in physical function and pain relief [27]. Such findings further support the rehabilitative effects of TCC for treating NLBP patients.

Accumulating evidence indicates that exercise intervention programs can help individuals with NLBP improve their quality of life, reduce pain and possibly avoid surgery. Most previous studies have observed benefits of CST programs [28,29] but the functional differences among different types of core stabilization exercises have rarely been investigated. Moreover, very few studies have focused on TCC (with an emphasis on trunk stability and a typical type of core stability) as a training exercise for NLBP. Notably, aging individuals with NLBP have also experienced a decline in neuromuscular function in the lower extremities. Thus, in this paper, we tested the potential beneficial effects of TCC for alleviating pain among aging adults with NLBP, specifically to determine whether TCC was effective in improving neuromuscular function. 

## 2. Methods

### 2.1. Participants

Forty-three Chinese community-dwellers were recruited in this study. Participants were only included if they were: (1) 55+ years old; (2) officially defined as NLBP; (3) able to move freely without any assistive technology; and (4) not involved in any TCC training program in the past three months. We excluded those who: (1) had scores above 8 on the Visual Analogue Scale (VAS); (2) had any pathology (e.g., infections, tumors or rheumatoid arthritis) causing lower back pain; (3) had serious liver, heart, lung, renal insufficiency or tumor; (4) had history of mental illness or cerebrovascular disease; (5) had neurological disease or skeletal muscle degenerative disease; or (6) had been exercising regularly. Our study protocol was approved by the University’s research ethics committee. Prior to starting the intervention, the study trial was registered at the Chinese Clinical Trial Registry (Registration number: ChiCTR-TRC-12002244). Procedures were implemented in accordance with the ethical standards of the Helsinki Declaration. Each participant signed an informed consent before testing began. 

**Figure 1:**
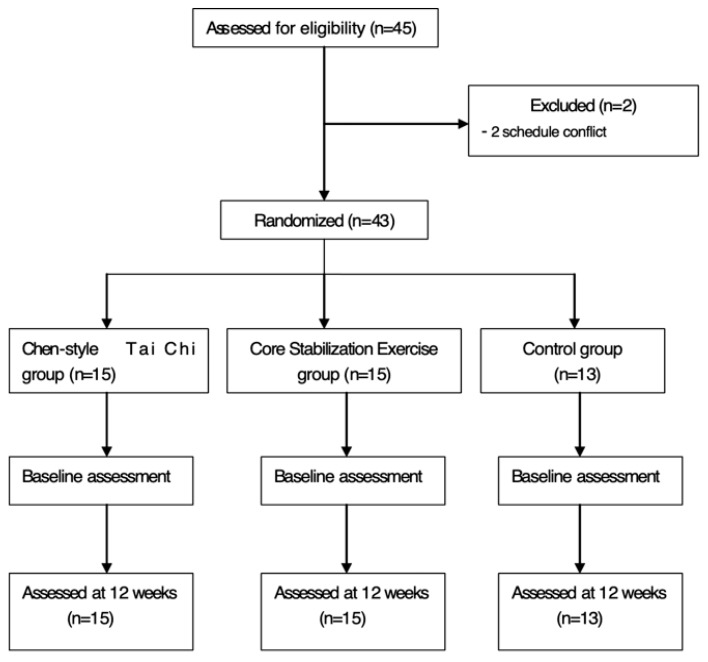
Displaying participant recruitment, randomization, outcome assessment and intervention.

Participants were randomly assigned to one of three groups using a computer-generated random-number sequence. The TCC group included fifteen participants (mean age = 58.13 ± 5.38 years; VAS baseline score = 5.67 ± 0.84; men and 11 women). Fifteen participants (mean age = 58.4 ± 5.08 years; VAS baseline score = 5.67 ± 0.72; VAS baseline score = 5.85 ± 0.89; 4 mem and 11 women) were assigned into the CST group, while the remaining three men and ten women (mean age = 60.67 ± 2.58 years; 3 men and 10 women) were assigned into the control group. Participants in the TCC and CST groups attended the designated exercise programs three times per week, with each session lasting 60 min per time for 12-week. The control group was asked to maintain their normal daily activities. Figure 1 presents the process of this experiment.

### 2.2. Exercise Intervention and Control Condition

Participants in the TCC group learned movements of the modified version of Chen-style TCC. Detailed information about the selected movements and training mode (frequency, intensity and progression) can be found in a previous study [30]. Each TCC session was held for a duration of 60 min, three times per week for 12 weeks. In the first two weeks, each session began with a 10 min warm-up (e.g., joint exercise and stretching), followed by 40 min on TCC principles (e.g., breathing technique and individual movement pattern) and then 10 min cool-down exercise. For the remaining 10 weeks, time allocation in each session remained similar but the training focus was different because the 40 min of TCC had greater emphasis on combinations of individual movements, coordinated with breathing control, body awareness and mental relaxation. An evidence-based CST program [30] was used in the present study and was designed based on the use of a Swiss ball to improve trunk muscle stability. Furthermore, there are six series of classical movements in this training plan: Glute Bridge Exercise, Single Leg Bridge Exercise, Bridge and Double Knee Flex, Single Leg Bridge and Double Knee Flex, Reverse Bridge Exercise, Reverse Bridge and Hip and Knee Flex [30]. During the intervention period, the CST program was administered by a certified physical therapist. Weekly training frequency was the same as the TCC program, while each session (same time allocation) lasted 60 min. Participants in the control group were asked to maintain their normal daily activities. 

### 2.3. Outcome Measures

All outcome assessments were performed at baseline and at the end of the trial (12 weeks), by trained assessors who were blinded to group assignment. The patient-based VAS was used to measure the level of perceived pain. Neuromuscular function was measured via the Biodex System 3 Isokinetic Dynamometer (Biodex Medical Systems, Shirley, NY, USA). Before each test, participants were asked to attend the instructor-led warm-up of five minutes, including sub-maximal exercise. 

#### 2.3.1. Pain Measured by the Visual Analog Scale 

The VAS is a visually accessible horizontally-oriented scale of 10 cm in length, with markings from “no pain” on the far left to “worst possible pain” on the far right. Participants were asked to mark points on the line to reflect their level of perceived pain of their current state.

#### 2.3.2. Neuromuscular Function Assessment

In the present study, we used the Biodex System 3 Isokinetic Dynamometer to measure lower-limb neuromuscular function. Furthermore, parameters of neuromuscular function included peak torque, peak torque to weight ratio and endurance. Each participant sat on the testing chair with the knee and hip in a 90 degrees position; straps were used to wrap around their thighs and ankles in order to minimize movements of the knee, respectively. For the knee joint, the angular velocities were set to 60°/s and 180°/s. Participants were asked to do five maximum concentric contractions for knee extensors and flexors at the two angular velocities. The peak torque, peak torque/weight of the flexor and extensor of knee and muscle endurance were recorded [30]. In addition, the angular velocity of 60°/s was used only for the ankle joint while the testing procedure remained same. 

### 2.4. Statistical Analysis

For each of the 37 features related to neuromuscular function and VAS in our interests, we calculated the change scores from the baseline to Week 12 and then performed multiple linear regression analysis for each feature, with the change score as dependent variable and the following variables as independent variables: two indicator variables GROUP_TC and GROUP_CR for grouping information (i.e., GROUP_TC = 1 stands for the TCC group and GROUP_CR = 1 stands for the CST group) ), an indicator variable GENDER (GENDER = 0 stands for female and GENDER = 1 stands for male), HEIGHT (in CM) and WEIGHT(in KG). The aim of this regression analysis was to test if group assignment significantly alters the average change score of each feature related to neuromuscular function and VAS, while adjusting for gender, height and weight. We tested if the regression parameters associated with GROUP_TC and GROUP_CR are equal to 0 respectively at a significance level 0.05, that is, if for one feature, the *p*-value corresponding to the regression parameter associated with GROUP_TC is less than 0.05, then it indicates a significant change of scores on average between the TC group and the control group, adjusting for other potential confounders. Similarly, if the *p*-value corresponding to the regression parameter associated with GROUP_CR is less than 0.05, then it indicates a significant change of scores on average between the CST group and the control group, adjusting for potential confounders. We performed the multiple linear regression analysis using the lm function in R (Austria, Non-profit Organization) [31]. 

## 3. Results

A total of 43 eligible patients with NLBP were included in our data analyses. No significant differences for demographic data was observed among the TCC, CST and control groups (ps > 0.05). Ratio of male to female participants was relatively equivalent among TCC (male = 4 and female 11), CST (male = 4 and female =11) and the control group (male = 3 and female =10). The average age of each group (TCC = 58.13 ± 5.38 years; CST = 58.4 ± 5.08 years; control group = 60.67 ± 2.58 years) was approximately 58 years. We have separately analyzed data on body weight (TCC = 58.93 ± 9.93 kg; CST = 63.33 ± 9.08 kg; control group = 63.47 ± 12.05 kg) and height (TCC = 159.53 ± 7.24 cm; CST = 162.53 ± 8.21 cm; control group = 163.47 ± 12.05 cm) in the present study.

For the VAS, we observed statistically significant differences between TCC and control groups (*p* < 0.01) and between CST and control groups (*p* < 0.01), as reported in a previous study [32]. For the knee joint, we only observed statistically significant differences on the endurance of left extension at a speed of 60°/s between TCC and control groups (*p* < 0.01) and between CST and control groups (*p* < 0.01). For the ankle joint, a statistically significant difference between CST and control groups was observed on the peak torque of left dorsiflexion (*p* < 0.05) and the endurance of left plantar flexion at a speed of 60°/s (*p* < 0.05). In addition, we observed a statistically significant difference between TCC and control groups on the endurance of right plantar flexion (*p* < 0.05). All mentioned-above statistically significant data are presented in Table 1, while non-statistically significant data are considered as supplementary data (Tables S1 and S2). 

## 4. Discussion 

The primary aim of the present study was to investigate the effects of TCC versus CST on neuromuscular function of the lower-limb among aging individuals with NLBP, while their effects on pain was demonstrated in our previous study [33]; TCC (baseline = 5.67 ± 0.81 and Week 12 = 3.47 ± 0.99) seems to be superior to the CST (baseline = 5.67 ± 0.72 and Week 12 = 4.27 ± 0.79). We found that both TCC and CST are effective in improving several parameters of neuromuscular functions in the lower limbs, as compared to control group. Detailed information about the study results will be discussed in the narrative that follows. 

### 4.1. Neuromuscular Function in the Knee 

It is well established that biological aging is associated with deleterious changes in neuromuscular function (e.g., muscle contractile properties and motor nerves) [34,35]. These impairments in muscle endurance and strength can result in reduced physical function, as well as reduce functional independence among individuals with NLBP [36,37]. More studies, therefore, need to be conducted to determine the rehabilitative effects of readily available and cost-effective approaches for pain reduction and enhancement in neuromuscular function in this vulnerable population. In the present study, we observed that both TCC and CST had protective effects on the endurance of left extension at a speed of 60°/s, as compared to the control group. Furthermore, when further evaluating the three individual groups at baseline and Week 12, only TCC showed positive improvement from baseline (0.98 ± 0.22) to Week 12 (1.1 ± 0.32), whereas decline in this outcome was observed in both CST (baseline = 1.01 ± 0.55 and Week 12 = 0.94 ± 0.11) and the control group (baseline = 0.95 ± 0.26 and Week 12 = 0.8 ± 0.28) after the 12-week intervention period; CST had less reduction in this outcome than the control group. Collectively, our study results indicate that TCC may improve the muscular endurance of left extension at a speed of 60°/s, while CST may attenuate the process of degeneration of this neuromuscular parameter. This positive finding may be attributed to the movement feature of TCC. For instance, backward sitting is performed through the entire TCC form and it involves knee extension with toe-off to support part of the body weight. With the 12-week TCC training, muscular endurance showed marginal improvements. A positive trend occurred in other parameters of the muscular endurance in TCC but did not reach statistical significance. Although speculative, perhaps these null findings are attributed to statistical power and thus, future studies employing larger sample sizes are warranted. 

### 4.2. Neuromuscular Function in the Ankle

The ankle is one of the most frequently injured joints, particularly in the aging population and it has a complex mechanical and anatomical structure [38]. Frequent injuries in the ankle joints can be attributed to rapid declines in muscular strength and endurance as people age [39]. Thus, identifying effective intervention programs are critical because it may help this aging population prevent injuries and perform normal physical activities [40]. Technology-based training has been applied in the majority of the previous studies on this topic but notably, this requires expensive equipment and may be difficult to implement in the community [41]. Exercise-based program that can improve muscular strength and endurance of the ankle joints should be recommended. In the present study, the CST improved the peak torque of left dorsiflexion (*p* < 0.05) and the endurance of left plantar flexion at a speed of 60°/s. These positive findings are possibly associated with movements in the CST program that involves dorsiflexion and plantar flexion of the ankle; when the feet are on the Swiss ball, it requires plantar flexion in order to keep the joints (shoulders, hip, knee and ankle) in alignment, while controlling the core muscle. When other movements involve the shoulders on the Swiss ball, this requires the feet to be placed on the ground. To keep the trunk flat in the air, the feet need to fight against the floor to generate sufficient force, which can strengthen the dorsiflexors. In addition, in the TCC group, we also observed improvements in endurance of the right plantar flexion at a speed of 60°/s, which is inconsistent with our previous study [32] investigating the effects of TCC on the strength and endurance of the bilateral ankle dorsiflexors and plantar flexors at a speed of 30°/s. This inconsistent finding may be partially explained by two factors. First, while Yang-style (constantly slow movement) in the previous study was used, we specifically considered Chen-style TCC as the intervention program in the present study because it involves more dynamic and powerful movements in the lower extremities. The intensity of Yang-style TCC may not be enough to improve this outcome. Second, the total time spent in TCC training is different between two studies; 1440 min (2 sessions × 45 min per week for 16 weeks) in Yang-style TCC [31] and 2160 min (3 sessions × 6 min per weeks for 12 weeks) in Chen-style TCC. Thus, 1440 min may not be enough to improve the flexion of the ankle [31]. Future studies need to take into account these factors. 

### 4.3. Strengths and Limitations

This is the first study investigating the effects of Chen-style TCC versus evidence-based CST program on lower-limb neuromuscular function in aging individuals with lower back pain. Authors of the present study used a rigorous randomized controlled design along with blinding of assessors. Other strengths involved use of a qualified TCC instructor, certified physical therapist and an objective assessment of the neuromuscular function throughout the entire intervention period. Some study limitations must be acknowledged as follows. First, given that this study included a relatively small sample, we did not analyze the relationship between pain and parameters of neuromuscular function in TCC and CST training groups, respectively, based on the mean change score (from baseline to Week 12). Thus, it still remains unclear whether or not improvements in the parameters of neuromuscular functions might be attributed to reduced pain. Improvement trends in some parameters of neuromuscular function were observed in this study but did not reach statistical significance, which might be partially explained by the relatively small sample size. Second, because it is unrealistic to blind participants to the exercise intervention, it cannot be determined whether or not participants in the two experimental groups had greater expectations about their intervention effects as compared to the non-active control group, which could contaminate the study results. Third, a lack of a medical diagnostic report may have influenced our ability to determine the precise nature of the participant’s actual health status. For instance, chronic neurological or musculoskeletal disorders are difficult to determine by participants themselves but could significantly affect neuromuscular function. 

## 5. Conclusions

The results of the present study suggest that both Chen-style TCC and CST have protective effects on neuromuscular function in aging individuals with NLBP, while alleviating non-specific chronic pain. In the present study, Chen-style TCC had a significantly greater effect on the muscular endurance of left knee extension in comparison to the evidence-based CST program. Future studies with large sample sizes are needed to confirm our findings. 

## Figures and Tables

**Table 1:** Visual Analogue Scale (VAS), neuromuscular function of knee and ankle at baseline and 12 weeks in Tai Chi Chuan group, Core Stability group and Control group.

Parameters	Tai Chi Chuan		Core Stability Training		Control	
	Baseline	Week 12	Baseline	Week 12	Baseline	Week 12
VAS	5.67 ± 0.81	3.47 ± 0.99 **	5.67 ± 0.72	4.27 ± 0.79 ^△△^	5.85 ± 0.89	5.85 ± 0.8
Knee						
Endurance: (60°/s)						
left extension	0.98 ± 0.22	1.1 ± 0.32 **	1.01 ± 0.55	0.94 ± 0.11 ^△△^	0.95 ± 0.26	0.8 ± 0.28
Ankle						
PT (Nm): 60°/s						
left dorsiflexion	13.99 ± 7.38	20.43 ± 4.83	12.1 ± 4.11	15.27 ± 5.1 ^△^	14.23 ± 9.41	16.9 ± 4.49
Endurance: (60°/s)						
right plantar flexion	2.10 ± 3.1	1.39 ± 0.78 *	1.08 ± 0.75	1.32 ± 0.84	3.42 ± 7.39	0.96 ± 0.37
left plantar flexion	1.01 ± 0.36	1.36 ± 0.77	0.92 ± 0.24	1.29 ± 0.92 ^△^	1.51 ± 1.46	0.8 ± 0.23

Note: ** = Tai-Chi Chuan group versus control group of the Week 12-minus-baseline assessment at the threshold of *p* < 0.01; ^△△^ = Core Stability Training group versus control group of the Week 12-minus-baseline assessment at the threshold of *p* < 0.01. * = Tai Chi Chuan versus control group of Week 12-minuts-baseline at the threshold of *p* < 0.05; ^△^ = Core Stability Training versus control of Week 12-minus-baseline assessment at the threshold of *p* < 0.05.

## Supplementary Materials

The following are available online at https://www.mdpi.com/1010-660X/55/3/60/s1, Table S1: VAS, neuromuscular function of knee at baseline and 12 weeks in Tai Chi Chuan group, Core Stability group and Control group., Table S2: Neuromuscular function of ankle at baseline and 12 weeks in Tai Chi Chuan group, Core Stability Training group, and Control group.

## References

[1] Golob A.L., Wipf J.E. (2014). Low back pain. Med. Clin. North Am..

[2] National Institute of Health (2018). Low Back Pain Fact Sheet. https://www.ninds.nih.gov/Disorders/Patient-Caregiver-Education/Fact-Sheets/Low-Back-Pain-Fact-Sheet.

[3] Centers for Disease Control and Prevention (2018). The National Institute for Occupational Safety and Health-Low Back Pain. https://www.cdc.gov/niosh/topics/nhis/data2015.html.

[4] Balagué F., Mannion A.F., Pellisé F., Cedraschi C. (2012). Non-specific low back pain. Lancet.

[5] Leinonen V., Määttä S., Taimela S., Herno A., Kankaanpää M., Partanen J., Kansanen M., Hänninen O., Airaksinen O. (2002). Impaired lumbar movement perception in association with postural stability and motor-and somatosensory-evoked potentials in lumbar spinal stenosis. Spine.

[6] Leinonen V., Kankaanpää M., Luukkonen M., Hänninen O., Airaksinen O., Taimela S. (2001). Disc herniation-related back pain impairs feed-forward control of paraspinal muscles. Spine.

[7] Tsao H., Galea M.P., Hodges P.W. (2008). Reorganization of the motor cortex is associated with postural control deficits in recurrent low back pain. Brain.

[8] Lamoth C.J., Meijer O.G., Daffertshofer A., Wuisman P.I., Beek P.J. (2006). Effects of chronic low back pain on trunk coordination and back muscle activity during walking: Changes in motor control. Eur. Spine J..

[9] Keeley P., Creed F., Tomenson B., Todd C., Borglin G., Dickens C. (2008). Psychosocial predictors of health-related quality of life and health service utilisation in people with chronic low back pain. Pain.

[10] Parthan A., Evans C.J., Le K. (2006). Chronic low back pain: Epidemiology, economic burden and patient-reported outcomes in the USA. Expert Rev. Pharm. Outcomes Res..

[11] Chou R., Deyo R., Friedly J., Skelly A., Hashimoto R., Weimer M., Fu R., Dana T., Kraegel P., Griffin J. (2017). Nonpharmacologic Therapies for Low Back Pain: A Systematic Review for an American College of Physicians Clinical Practice Guideline. Ann. Intern. Med..

[12] Wang X.Q., Zheng J.J., Yu Z.W., Bi X., Lou S.J., Liu J., Cai B., Hua Y.H., Wu M., Wei M.L. (2012). A meta-analysis of core stability exercise versus general exercise for chronic low back pain. PLoS ONE.

[13] Natour J., Cazotti L.D.A., Ribeiro L.H., Baptista A.S., Jones A. (2015). Pilates improves pain, function and quality of life in patients with chronic low back pain: A randomized controlled trial. Clin. Rehabil..

[14] Rydeard R., Leger A., Smith D. (2006). Pilates-based therapeutic exercise: Effect on subjects with nonspecific chronic low back pain and functional disability: A randomized controlled trial. J. Orthop. Sports Phys. Ther..

[15] Marshall P.W., Murphy B.A. (2006). Evaluation of functional and neuromuscular changes after exercise rehabilitation for low back pain using a Swiss ball: A pilot study. J. Manip. Physiol. Ther..

[16] Zou L., Sasaki J.E., Wei G.X., Huang T., Yeung A.S., Neto O.B., Chen K.W., Hui S.C. (2018). Effects of Mind–Body Exercises (Tai Chi/Yoga) on Heart Rate Variability Parameters and Perceived Stress: A Systematic Review with Meta-Analysis of Randomized Controlled Trials. J. Clin. Med..

[17] Zou L., Yeung A., Li C., Wei G., Chen K., Kinser P., Chan J., Ren Z. (2018). Effects of meditative movements on major depressive disorder: A systematic Review and meta-analysis of randomized controlled trials. J. Clin. Med..

[18] Zou L., Yeung A., Li C., Chiou S., Zeng N., Tzeng H. (2018). Effects of mind-body movement on balance function in stroke survivors: A meta-analysis of randomized controlled trials. Int. J. Env. Res. Public Health.

[19] Zou L., Sasaki J., Zeng N., Wang C., Sun L.A. (2018). Systematic Review with Meta-Analysis of Mindful Exercises on Rehabilitative Outcomes among post-stroke patients. Arch. Phys. Med. Rehabil..

[20] Zou L., Wang H., Xiao Z., Fang Q., Zhang M., Li T., Du G., Liu Y. (2017). Tai chi for health benefits in patients with multiple sclerosis: A systematic review. PLoS ONE.

[21] Zou L., Han J., Tsang W., Yeung A., Hui S.S., Tsang W.W.N., Ren Z., Wang L. (2018). Effects of Tai Chi on lower limb proprioception in adults aged over 55: A systematic review ad meta-analysis. Arch. Phys. Med. Rehabil..

[22] Wang C., Collet J.P., Lau J. (2004). The effect of Tai Chi on health outcomes in patients with chronic conditions: A systematic review. Ann. Intern. Med..

[23] Hall A.M., Maher C.G., Lam P., Ferreira M., Latimer J. (2011). Tai chi exercise for treatment of pain and disability in people with persistent low back pain: A randomized controlled trial. Arthritis Care Res..

[24] Zou L., Wang H., Yu D. (2017). Effect of a long-term modified Tai Chi-based intervention in attenuating bone mineral density in postmenopausal women in southeast China: Study protocol for a randomized controlled trial. Clin. Trials Degener. Dis..

[25] Zou L., Wang C., Tian Z., Wang H., Shu Y. (2017). Effect of Yang-Style Tai Chi on Gait Parameters and Musculoskeletal Flexibility in Healthy Chinese Older Women. Sports.

[26] Lou L., Zou L., Fang Q., Wang H., Liu Y., Tian Z., Han Y. (2017). Effect of Taichi Softball on Function-Related Outcomes in Older Adults: A Randomized Control Trial. Evid-Based Complement. Altern. Med..

[27] Morone N.E., Greco C.M., Weiner D.K. (2008). Mindfulness meditation for the treatment of chronic low back pain in older adults: A randomized controlled pilot study. Pain.

[28] Gatantino M.L., Bzdewka T.M., Eissler-Rnsso J.L., Holbrook M.L., Mogck E.P., Geigle P., Farrar J.T. (2004). The impact of modified Hatha yoga on chronic low back pain: A pilot study. Altern. Ther. Health Med..

[29] Tilbrook H.E., Cox H., Hewitt C.E., Kang’ombe A.R., Chuang L.H., Jayakody S., Aplin J.D., Semlyen A., Trewhela A., Watt I. (2011). Yoga for chronic low back pain: A randomized trial. Ann Intern Med.

[30] Liu J., Wang X.Q., Zheng J.-J., Pan Y.J., Hua Y.H., Zhao S.M., Shen L.Y., Fan S., Zhong J.G. (2012). Effects of Tai Chi versus Proprioception Exercise Program on Neuromuscular Function of the Ankle in Elderly People: A Randomized Controlled Trial. Evid-Based Complement. Altern. Med..

[31] R Core Team (2019). R: A Language and Environment for Statistical Computing.

[32] Wang X.Q., Zheng J.G., Xia B., Liu J. (2012). Effect of core stability training on patients with chronic low back pain. Health Med..

[33] Liu J., Yeung A., Xiao T., Tian X.P., Kong Z.W., Zou L., Wang X.Q. (2019). Chen-style Tai Chi for individuals with chronic non-specific low back pain over: A randomized controlled trial. Int. J. Env. Res. Public Health.

[34] Romero-Arenas S., Martínez-Pascual M., Alcaraz P.E. (2013). Role of muscle loss in the age-associated reduction in VO2max. Aging Dis..

[35] Izquierdo M., Häkkinen K., Antón A., Garrues M., Ibañez J., Ruesta M., Gorostiaga E.M. (2001). Maximal strength and power, endurance performance, and serum hormones in middle-aged and elderly men. Med. Sci. Sports Exerc..

[36] Izquierdo M., Häkkinen K., Ibanez J., Antón A., Garrués M., Ruesta M., Gorostiaga E.M. (2003). Effects of strength training on submaximal and maximal endurance performance capacity in middle-aged and older men. J. Strength Cond. Res..

[37] Snijders T., Verdijk L.B., van Loon L.C. (2009). The impact of sarcopenia and exercise training on skeletal muscle satellite cells. Ageing Res. Rev..

[38] Izquierdo M., Ibanez J., Gorostiaga E.M., Garrues M., Zuñiga A., Antón A., Larrión J.L., Häkkinen K. (1999). Maximal strength and power characteristics in isometric and dynamic actions of upper and lower extremities in middle-aged and older med. Acta Physiol. Scand..

[39] Jeon K.K., Kim T.Y., Lee S.H. (2015). The effects of a strategic strength resistance exercise program on the isokinetic muscular function of the ankle. J. Phys. Ther. Sci..

[40] Davies C.T., Thomas D.O., White M.J. (1986). Mechanical properties of young and elderly human muscle. Acta Med. Scand..

[41] Lan C., Lai J., Chen S.Y., Wong M.K. (2000). Tai Chi Chuan to improve muscular strength and endurance in elderly individuals: A pilot study. Arch. Phys. Med. Rehabil..

