# Supplementary material for: The Effects of Tai Chi Chuan Versus Core Stability Training on Lower-Limb Neuromuscular Function in Aging Individuals with Non-Specific Chronic Lower Back Pain
Source: Medicina (Kaunas). 2019 Mar 3;55(3):60. doi: 10.3390/medicina55030060 (PMC6473361; doi:10.3390/medicina55030060)
Supplement: Supplementary file 1 [file medicina-55-00060-s001.pdf]

Table S1. VAS, neuromuscular function of knee at baseline and 12 weeks in Tai Chi Chuan group, Core Stability group and Control group.

Parameters	Tai Chi Chuan		Core Stability Training		Control	
	**Baseline**	**Week 12**	**Baseline**	**Week 12**	**Baseline**	**Week 12**
PT (Nm): 60°/s						
right extension	93.14 ± 24.86	100.17 ± 28.07	74.94 ± 16	81.21 ± 22.36	68.05 ± 14.35	70.16 ± 14.59
left extension	56.7 ± 18.97	77.2 ± 18.21	55.12 ± 18.14	59.34 ± 20.13	50.14 ± 14.87	54.35 ± 15.47
right flexion	77.77 ± 23.92	91.36 ± 36.81	74.51 ± 21.69	79.9 ± 23.12	68.22 ± 14.2	75.3 ± 14.66
left flexion	51.95 ± 12.06	65.66 ± 16.58	46.63 ± 17.33	55.66 ± 17.53	48.22 ± 14.2	51.23 ± 14.1
PT/weight (Nm/kg): 60°/s						
right extension	158.18 ± 27.87	164.34 ± 29.92	117.62 ± 22.07	126.32 ± 26.1	111.37 ± 32.67	116.3 ± 27.73
left extension	96.29 ± 22.1	113.68 ± 23.37	85.23 ± 21.03	91.11 ± 23.95	99.36 ± 18.64	107.06 ± 17.39
right flexion	133.58 ± 34.5	153.36 ± 44.4	117.13 ± 34.1	123.3 ± 23.61	109.59 ± 28.27	121.1 ± 29.63
left flexion	88.68 ± 13.43	95.13 ± 17.79	71.86 ± 21.18	85.28 ± 17.6	66.15 ± 24.6	71.93 ± 18.58
Endurance: (60°/s)						
right extension	0.9 ± 0.21	1.24 ± 0.3	1.11 ± 0.44	1.04 ± 0.16	1.02 ± 0.4	0.88 ± 0.19
Right flexion	1.08 ± 0.33	1.45 ± 0.52	0.98 ± 0.37	1.04 ± 0.41	1.08 ± 0.3	0.93 ± 0.2
left flexion	0.91 ± 0.35	1.17 ± 0.39	0.79 ± 0.26	1.02 ± 0.26	0.99 ± 0.45	1.15 ± 0.67
PT (Nm): 180°/s						
right extension	53.7 ± 20.9	53.2 ± 18.7	45.55 ± 12.78	52.47 ± 16.86	46.14 ± 11.13	49.53 ± 13.43
left extension	41.8 ± 10.91	43.6 ± 10.09	39.22 ± 10.44	45 ± 13.43	37.23 ± 6.68	39.58 ± 10.23
right flexion	49.5 ± 19.23	52.64 ± 19.05	40.4 ± 10.74	49.64 ± 14.59	42.93 ± 9.41	46.23 ± 10.99
left flexion	40.07 ± 9.04	43.1 ± 2.51	33.41 ± 10.3	43.54 ± 13.17	38.39 ± 10.84	40.9 ± 10.46
PT/weight (Nm/kg):180°/s						
right extension	82.29 ± 19.87	86.48 ± 23.06	71.43 ± 17.35	81.35 ± 19.8	73.53 ± 19.15	78.92 ± 23.46
left extension	71.02 ± 12.37	74.88 ± 14.0	61.73 ± 15.76	69.36 ± 14.6	59.7 ± 14.55	62.96 ± 16.24
right flexion	70.28 ± 23.06	77.08 ± 22.31	61.55 ± 17.14	76.26 ± 13.09	68.15 ± 13.93	73 ± 13.72
left flexion	68.38 ± 10.05	74.32 ± 14.07	51.8 ± 12.89	67.02 ± 13.84	61.03 ± 17.14	65.03 ± 15.37
Endurance: (180°/s)						
right extension	1.44 ± 2.4	0.77 ± 0.35	1.22 ± 0.47	0.87 ± 0.3	1.47 ± 2.13	0.8 ± 0.24
left extension	0.84 ± 0.3	0.9 ± 0.56	1.0 ± 0.46	0.78 ± 0.12	0.76 ± 0.25	0.79 ± 0.12
Right flexion	0.82 ± 0.2	1.02 ± 0.33	1.01 ± 0.39	1.23 ± 0.44	0.82 ± 0.2	0.98 ± 0.61
left flexion	1.32 ± 1.47	1.26 ± 1.7	0.88 ± 0.37	1.08 ± 0.35	0.91 ± 0.2	0.81 ± 0.22

Table S2. Neuromuscular function of ankle at baseline and 12 weeks in Tai Chi Chuan group, Core Stability Training group, and Control group.

Parameters	Tai Chi Chuan		Core Stability Training		Control Group	
	Baseline	Week 12	Baseline	Week 12	Baseline	Week 12
PT (Nm): 60°/s						
right plantar flexion	17.09 ± 8.24	24.27 ± 7.96	14.08 ± 4.61	18.02 ± 4.38	16.13 ± 6.95	16.7 ± 5.18
left plantar flexion	13.37 ± 5.6	18.67 ± 6.25	12.19 ± 3.79	15.1 ± 5.62	11.35 ± 5.63	12.9 ± 4.68
right dorsiflexion	19.24 ± 7.36	26.66 ± 7.71	18.15 ± 6.44	21.62 ± 5.13	19.39 ± 4.98	20.36 ± 5.59
PT/weight (Nm/kg): 60°/s						
right plantar flexion	26.57 ± 7.63	36.18 ± 9.43	22.26 ± 7.74	28.05 ± 5.65	26.66 ± 13.22	26.97 ± 9.61
left plantar flexion	23.19 ± 9.59	30.64 ± 9.25	19.1 ± 5.78	26.6 ± 9.34	18.08 ± 8.7	20.43 ± 8.07
right dorsiflexion	33.62 ± 13.34	45.32 ± 10.64	28.67 ± 9.89	33.8 ± 6.71	31.64 ± 11	33.18 ± 11.74
left dorsiflexion	24.52 ± 12.93	34.16 ± 7.34	18.98 ± 5.74	31.43 ± 8.37	19.43 ± 7.69	22.89 ± 6.22
Endurance: (60°/s)						
right dorsiflexion	0.83 ± 0.3	1.07 ± 0.37	1.14 ± 0.42	1.0 ± 0.22	1.61 ± 1.47	1.05 ± 0.23
left dorsiflexion	0.75 ± 0.31	1.00 ± 0.39	1.71 ± 1.91	1.39 ± 1.71	3.49 ± 7.38	0.87 ± 0.19
